# Determining the HPV vaccine schedule for a HIV-infected population in sub Saharan Africa, a commentary

**DOI:** 10.1186/s12985-018-1039-y

**Published:** 2018-08-16

**Authors:** Sonia Menon, Rodolfo Rossi, Mbabazi Kariisa, Steven Callens

**Affiliations:** 1 International Centre for Reproductive Health (ICRH), Department of Obstetrics and Gynaecology, Ghent University, De Pintelaan 185 P3, 9000 Ghent, Belgium; 2 International Committee of Red Cross, Geneva, Switzerland; 3 March of Dimes Foundation, White Plains, New York, USA; 4 Department of Internal Medicine & Infectious diseases, University Hospital, Ghent, Belgium

**Keywords:** Vaccine schedule, Sub Saharan Africa, HIV

## Abstract

**Background:**

Epidemiological studies have established human papillomavirus (HPV) infection as the central cause of invasive cervical cancer (ICC) and its precursor lesions. HIV is associated with a higher prevalence and persistence of a broader range of high-risk HPV genotypes, which in turn results in a higher risk of cervical disease. Recent WHO HPV vaccination schedule recommendations, along with the roll out of HAART at an earlier CD4 count within the female HIV-infected population, may have programmatic implications for sub Saharan Africa. This communication identifies research areas, which will need to be addressed for determining a HPV vaccine schedule for this population in sub Saharan Africa. A review of WHO latest recommendations and the evidence concerning one-dose HPV vaccine schedules was undertaken.

**Conclusion:**

For females ≥15 years at the time of first dose and immunocompromised and/or HIV-infected, a 3-dose schedule (0, 1–2, 6 months) is recommended for all three vaccines. There is some evidence that there is similar protection against HPV 16 and 18 infection from a single vaccination than from two or three doses, however there is no cross protection conferred to other genotypes. There is a need for periodic prevalence studies to determine the vaccination coverage of bivalent, quadrivalent and nonavalent vaccine targeted oncogenic HPV genotypes in women with CIN 3 or ICC at national level. In light of the increasing number of sub Saharan HIV-infected girls initiating HAART at a CD4 count above 350 mm^3,^ there are a number of clinical, virological and public health research gaps to address before a tailored vaccine schedule can be established for this population.

## Background

Cervical cancer is the fourth most common malignancy with 528,000 new cases reported worldwide in 2015 [1]. In resource-poor countries, delayed diagnosis often makes cure impossible. Forty HPV genotypes have been identified, of which 14 high risk types (HR-HPV) are associated with the development of high-grade precancerous squamous intraepithelial lesions and subsequent invasive cervical cancer (ICC) [2]. Although most HPV infections clear without intervention within 1 year, certain high-risk HPV (HR-HPV) genotypes tend to persist and are consequently the chief risk factor for ICC onset [3].

Several sub Saharan countries have licensed and adopted the bivalent HPV vaccine (Cervarix™) that protects against HPV genotypes 16 and 18 and the quadrivalent vaccine (Gardasil™) that protects against HPV genotypes 6, 11, 16 and 18 [4]. The bivalent and quadrivalent prophylactic vaccine, which contain the genotypes responsible for about 70% of cervical cancers globally, constitute a breakthrough for primary prevention. Furthermore, the bivalent HPV vaccine offers substantial cross protection against HPV genotypes 31/33/45 as demonstrated in both clinical trials and confirmed in Scottish HPV surveillance study [5]. A new nonavalent vaccine containing HPV types, containing the most prevalent genotypes found in ICC worldwide 6, 11, 16, 18, 31, 33, 45, 52 and 58 [6] may have direct implications for cervical cancer incidence and prevention potentially preventing almost 90% of ICC cases worldwide [7]. The WHO recommends including the HPV vaccination in national immunization programs provided HPV represents a public health priority and vaccination is feasible and cost-effective [8].

HIV-positive women are particularly at risk for HPV infection and precancerous lesions, for whom, lesions are more aggressive, persistent and more likely to recur following treatment [9]. In primary cervical cancer prevention, there is another potentially effective weapon. Our recent systematic review of HAART effects on the presence of HPV, pre-malignant and malignant cervical lesions in Sub-Saharan Africa found evidence that duration of HAART, along with the CD4 count, may reduce the prevalence of HR-HPV [10].

This communication paper identifies research gaps for determining a vaccine schedule for HIV-infected girls in sub Saharan Africa. We explored WHO policy papers and guidelines concerning HPV vaccination schedules and examined the evidence for a one-dose regimen.

## Main text

### Target group and vaccination schedule

For the bivalent, quadrivalent and nonavalent vaccines, the vaccination schedule depends on the age and immunocompetency of the recipient. Girls < 15 years at the time of first dose: a 2-dose schedule (0, 6 months) is recommended. The two doses should ideally be separated by 6 to 12 months and the interval from the first dose should not be less than 5 months [11]. For women aged 15 years and older, and those immunocompromised and/or HIV-infected, receiving HAART or not, a 3-dose schedule (0, 1–2, 6 months) is recommended [11].

Whilst, studies have shown the bivalent, quadrivalent and nonavalent to be safe and immunogenic in HIV-infected women [12] with women with HIV RNA load > 10,000 copies/ml and or CD4 count < 200 cells having lower rates of seroconversion rates, there is to date no efficacy data from HIV-infected individuals in Sub Saharan Africa. Nor is the duration of adequate antibody titers to the vaccine associated HPV types established, which precludes a boosting vaccine dosing regimen from being established. A specific aspect to consider within the sub Saharan continent, where helminths are among the major public health problems [13], is the resultant dominant Th2 helper immune response [14]. In turn a dominant Th2 helper immune response may further diminish the necessary Th1 response in HIV infected female and as corollary, modify the potency of the vaccine [15].

Moreover, with HAART initiation at a higher CD4 count, seroprevalence studies should consider whether HIV-infected girls (9–14 years), who have initiated HAART at a CD4 count above 350 mm^3^ are immunologically non-inferior to immunocompetent girls having been administered the recommended two-dose schedule recommended for the bivalent, quadrivalent, and nonavalent HPV vaccine in that age group.

The impact of the bivalent or quadrivalent vaccine in the HIV-infected female population in sub-Saharan Africa will depend on the prevalence of HPV 16 and HPV 18. A recent meta-analysis (2016) on the distribution of pHR/HR HPV genotypes in HIV-infected women in Kenya reported a pooled estimated prevalence of HPV 16 and 18 of 61% in women with ICC [16], in presence of multiple HPV co-infections. Whilst HPV-16 and HPV-18 pose much higher cancer risks than any other HPV type and replacement by a nononcogenic type or an oncogenic type is not expected to have any major consequences in a general population [17], it is unclear what this shift in a HIV-infected population may entail. Given the wide spectrum of HPV genotypes reported in HIV-infected women, the threat of type replacement [18] by hitherto less prevalent HPV genotypes, including HPV 52, 56 along with the potentially high risk of HPV 53 after the successful elimination of HPV 16 and 18, underscores the importance of broader primary prevention programs.

### One-dose schedule

A study combining data from two large trials found similar protection against HPV 16 and 18 from a single bivalent vaccination as from the current two and three dose schedules [19]. Moreover, early findings from an Indian study indicate that a single dose of quadrivalent HPV vaccine is immunogenic and provides lasting protection against HPV 16 and 18 infections similar to the three and two dose vaccine schedules [20].

Apart from helping to overcome programmatic barriers in resource-poor settings, if HPV vaccines could be delivered as single dose, while retaining their efficacy against the most oncogenic HPV types 16 and 18, it may open a great opportunity to extend the reach of protection to more people [21].

However, protection against HPV types phylogenetically-related to HPV 16, HPV 31 and 33, and against HPV45, which is phylogenetically-related to HPV 18, probably attributable to cross-neutralizing antibodies, may be lower with alternate vaccine schedule as compared with the standard three dose regimen [22]. Despite the potential loss of cross-protection, a single dose may be sufficient to reduce the number of ICC worldwide by 70% [23]. It is unknown whether a one-dose HPV vaccination schedule can confer similar protection to HIV-infected women with a moderately reconstituted immune system. Should studies suggest that similar protection can be extended to women with moderately reconstituted immune systems, cost-effectiveness analyses must be undertaken to compare a one dose regimen to a two/three-dose vaccine regimen in HIV-infected girls/women based on the epidemiology of pHR/HR HPV genotypes within each context.

### Catch-up campaigns

In 2016, the WHO revised its position to recommend delivering vaccination to multiple age cohorts of girls aged 9–14 years in resource-poor settings in order to increase HPV vaccine uptake and bring forward the benefits of vaccination in the population [24], following the rapid effectiveness seen in many industrialized countries where HPV vaccine introduction has been extended to the age of 26.

Assuming affordable vaccine cost and knowledge of the local distribution of HPV genotypes, additional catch-up rounds may be beneficial to older HIV-infected girls, whose future access to cervical screening is uncertain and who are at risk of acquiring and transmitting multiple HPV co-infections. It has to be recognised, though, that vaccination campaigns are not meant to replace routine immunisation services if sufficient coverage is to be maintained [25] and that as female school enrolment in many countries drops after primary school, a school-based vaccination strategy may not be successful in capturing older vulnerable HIV-infected girls.

However, it is unclear whether, among women infected with HPV, the residual benefit of preventing infection with HPV types contained in the vaccine to which the women have not yet been exposed would be sufficient to warrant vaccination [26]. The lesser impact expected of the HPV vaccine in older HIV-infected women due to a potential higher risk of prior exposure to vaccine targeted genotypes, may be compensated by the potential of the HPV vaccine to prevent recurrence of CIN 2–3 among women treated for HSIL [27], possibly due to generated antibodies providing protection against new infection or reinfection from other areas of the genital tract with vaccine targeted types. Estimates for the incidence of disease recurrence after treatment vary widely from 25 to 55% at 12 months in HIV-infected women compared with 5–16% in HIV-negative women [28].

Prior to the implementation of a catch-up campaign, a cost-effectiveness study should be performed. This would entail comparing the number of cases of HSIL and cancer, with the associated costs of treatment and lives/quality of life lost, that would occur with screening alone, single cohort vaccination only, or single cohort plus catch up vaccination.

## Conclusion

The nonavalent vaccine may provide an incremental benefit beyond the current bivalent and quadrivalent vaccines, but in light of their current prohibitive prices, it will need to be determined whether resources should be allocated toward vaccinating more girls with either the bivalent or quadrivalent vaccine. In order to determine the potential cost effectiveness of the more expensive nonavalent vaccine within sub-Saharan countries, regular prevalence studies should be carried out to assess the vaccination coverage of bivalent, quadrivalent and nonavalent vaccine targeted oncogenic HPV genotypes in women with CIN 3 or ICC at national level. In addition, the cancer genesis potential of HPV genotypes excluded by the bivalent/quadrivalent vaccines will need to be elucidated in women who initiate HAART at a higher CD4 count. Furthermore, the potential of the HPV vaccines to prevent CIN 2/3 recurrence in HIV-infected women should be urgently examined.

Apart from determining the effectiveness of the HPV vaccine in sub Saharan HIV-infected women with a reconstituted immune system and concomitant polyparasitic infections, in light of the high prevalence of non-HPV 16 and 18 within this population, surveillance should be strengthened to monitor the risk of type replacement.

As in sub Saharan African, it is mostly rural women who present with ICC, a catch-up campaign should consider adopting a clinic-based vaccination strategy, one which would be based on an established infrastructure of rural HIV treatment program.

## Abbreviations

HAART: Highly active antiretroviral therapy
HIV: Human immunodeficiency virus
HPV: Human Papilloma virus
